# Scalable ion concentration polarization dialyzer for peritoneal dialysate regeneration

**DOI:** 10.1186/s12951-025-03294-1

**Published:** 2025-03-29

**Authors:** Wonseok Kim, Seongjun Hong, Kihong Kim, Sunhwa Lee, Dong Ah Shin, Seung Hee Yang, Jeongeun Lee, Kyunghee Kim, Kyoung Jin Lee, Woo Sang Cho, Hajeong Lee, Dong Ki Kim, Hee Chan Kim, Yon Su Kim, Jung Chan Lee, Gun Yong Sung, Sung Jae Kim

**Affiliations:** 1 Department of Electrical and Computer Engineering, Seoul National University, Seoul, 08826 Republic of Korea; 2 SOFT Foundry Institute, Seoul National University, Seoul, 08826 Republic of Korea; 3 Inter-University Semiconductor Research Center, Seoul National University, Seoul, 08826 Republic of Korea; 4 Division of Nephrology, Kangwon National University Hospital, Chuncheon, 24289 Republic of Korea; 5 Kidney Research Institute, Seoul National University Medical Research Center, Seoul, 03080 Republic of Korea; 6 Biomedical Research Institute, Seoul National University Hospital, Seoul, 08826 Republic of Korea; 7 Interdisciplinary Program in Bioengineering, Graduate School, Seoul National University, Seoul, 08826 Republic of Korea; 8 Institute of Medical and Biological Engineering, Seoul National University Medical Research Center, Seoul, 03080 Republic of Korea; 9 Major in Materials Science and Engineering, School of Future Convergence, Hallym University, Chuncheon, 24252 Republic of Korea; 10 Interdisciplinary Program of Nano-Medical Device Engineering, Graduate School, Hallym University, Chuncheon, 24252 Republic of Korea; 11 Integrative Materials Research Institute, Hallym University, Chuncheon, 24252 Republic of Korea; 12 Division of Nephrology, Department of Internal Medicine, Seoul National University Hospital, Seoul, 03080 Republic of Korea; 13 CHA Future Medicine Research Institute, Seongnam-si, 13488 Republic of Korea; 14 SNU Energy Initiative, Seoul National University, Seoul, 08826 Republic of Korea; 15 Department of Biomedical Engineering, Seoul National University College of Medicine, Seoul, 03080 Republic of Korea

## Abstract

A wearable artificial kidney (WAK) stands poised to offer dialysis treatment with maximal temporal and spatial flexibility for end-stage renal disease (ESRD) patients, while portability has not yet been achieved due to difficulties in portable purification. The ion concentration polarization (ICP), one of the nanoelectrokinetic phenomenon, has garnered substantial attention in the realm of portable purification applications, owing to its remarkable capacity for charge separation. In this work, scalable ICP dialyzer with 10,000-fold increase in throughput, was applied for peritoneal dialysate regeneration. First, the mechanism underpinning dialysate purification was corroborated based on micro-nanofluidics. Simultaneously, the electrochemical reactions utilized the complete decomposition of uncharged toxin (urea), achieving approximately 99% clearance, while the ICP phenomenon promoted the removal of positively charged toxin (creatinine), achieving approximately 30% clearance. Second, 3-D scalable ICP dialyzer was developed with a creation of micro-nanofluidic environment inside. Throughput scalability was demonstrated up to 1 mL/min with average approximately 30% toxins clearance. Ultimately, the 3-D ICP dialyzer was applied to assist peritoneal dialysis (PD) using a bilateral nephrectomy rat model. We demonstrated that regenerated dialysate successfully reduced in vivo toxicity, with average toxins removal ratio of approximately 30% per cycle. We believe that the integration of this scalable ICP dialyzer into the WAK holds tremendous potential for substantially enhancing the quality of life for individuals with ESRD.

**Graphical abstract:**

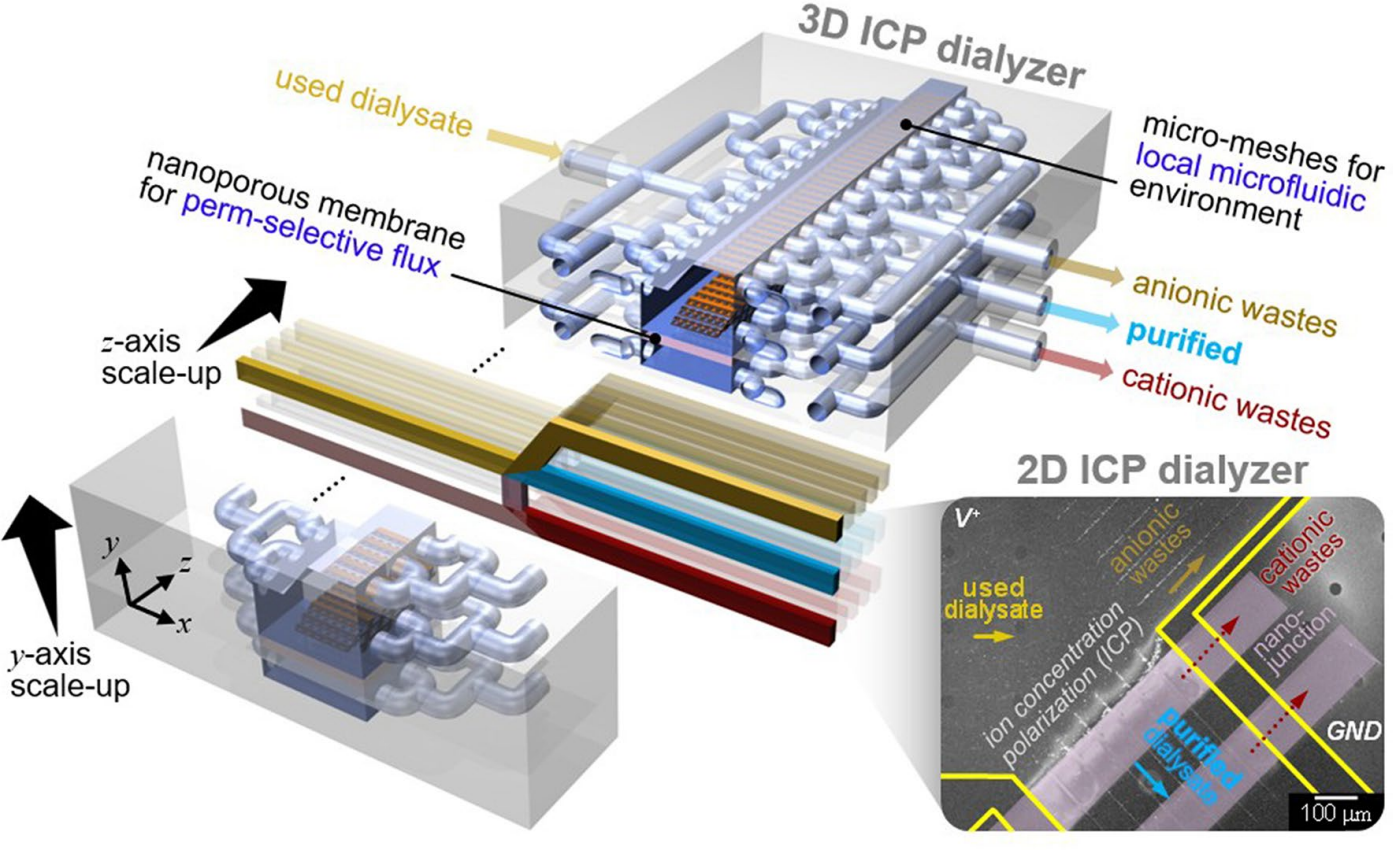

**Supplementary Information:**

The online version contains supplementary material available at 10.1186/s12951-025-03294-1.

## Introduction

End-stage renal disease (ESRD) signifies a condition characterized by permanent kidney failure, carrying a heightened risk of morbidity and mortality. The global population of individuals afflicted with ESRD is observing a notable escalation at an annual growth rate of 5–7%, resulting in an amplified economic burden [1]. Even with appropriate dialysis interventions, these patients confront cardiovascular mortality rates approximately 30 times higher than the general populace [2]. Adjusted mortality rates of ESRD patients amounted to 136 per 1,000 patient-years in recent United States Renal Data System (USRDS) annual report [3]. Furthermore, Medicare expenditures for ESRD patients incur the highest medical expenses per patient compared to other chronic diseases, amounting to USD 30.9 billion in the United States for the year 2013. Individuals grappling with ESRD are presented with the choices of kidney transplantation, hemodialysis (HD), or peritoneal dialysis (PD) as therapeutic modalities [4]. Kidney transplantation stands as the optimal choice due to its superior survival rates [5–7], enhanced quality of life, and cost-effectiveness [8–10], although challenges concerning donor availability remain unresolved. Those without available altruistic donor candidates often resort to HD or PD. The selection of the dialysis method hinges upon the patient’s medical condition and characteristics [11–13]. HD, a prevailing and effective renal replacement therapy, involves the utilization of advanced dialysis machines to meticulously extract waste products and excess fluids from the blood through a specialized dialysis membrane. This method, managed by specialized medical personnel, offers relative ease of application. Nevertheless, HD patients typically undergo dialysis sessions three times a week at a clinic, necessitating the removal of accumulated waste and fluids generated over a span of 2 or 3 days within a mere 4-hour timeframe. These recurrent fluctuations in volume status, electrolyte balance, and acid-base equilibrium engender adverse effects on cardiovascular health, contributing to elevated mortality rates.

PD employs the patient’s peritoneum, the lining of the peritoneal cavity, as a medium for blood filtration. Substances such as urea, creatinine, potassium, phosphate, and other uremic toxins in the blood are exchanged with a solution called dialysate over the course of 4 to 6 h. The patient is responsible for self-replacement of the used dialysate [14]. PD is particularly favored by younger and more active patients due to its manageable time and spatial demands during the dialysis process. It fundamentally emulates the natural kidney function of “continuous filtration”, granting patients more dietary freedom and aiding in the preservation of residual kidney function. In terms of outcomes, PD has demonstrated exceptional survival rates, especially during the initial 2 years after commencing dialysis [15–17], surpassing HD, even extending its benefits into the post-transplantation phase [18, 19].

However, despite its advantages, PD remains a less-preferred dialysis modality worldwide when compared to HD. This preference gap is attributed to PD’s inadequacies in effectively eliminating uremic toxins, its cumbersome and frequent self-exchange system employing unwieldy dialysate bags, and the occurrence of infectious and metabolic complications such as diabetes, obesity, and metabolic syndrome. These inconveniences could be addressed through the introduction of a portable peritoneal dialysate regeneration system. While certain mechanisms such as biochemical adsorption or physical filtration have been suggested, inherent limitations like clogging or frequent filter replacements have impeded further development and commercialization [20, 21].

In recent years, the field of water treatment has actively pursued distributed, small-capacity water treatment systems characterized by high energy efficiency, sustainable equipment costs, and minimized environmental impacts. Among various promising candidates, electrochemical techniques like electrodialysis (ED) [22, 23], electrodeionization (EDI) [24], and capacitive deionization (CDI) [25, 26] have gained attention due to their suitability for small-scale applications. Reverse osmosis (RO), the most prevalent method, is primarily suited for large-scale systems [27]. For example, to purify dialysate (~ 150 mM), RO must maintain a minimum pressure of 3.69 atmospheres as below calculation, which makes it unsuitable for a portable form.

$$\eqalign{{\rm{\pi }}\,{\rm{ = }} & {\rm{CRT = 0}}{\rm{.15 }}\left( {{\rm{M/L}}} \right){\rm{ \times 8}}{\rm{.41 (J/M \cdot K)}} \cr & {\rm{ \times 300 }}\left( {\rm{K}} \right){\rm{ = 378}}{\rm{.63 }}\left( {{\rm{J/L}}} \right){\rm{ }} \cr & {\rm{ = 374130 }}\left( {{\rm{pa}}} \right){\rm{ = 3}}{\rm{.69 }}\left( {{\rm{atm}}} \right) \cr} $$

where (C: molar concentration, R: ideal gas constant, T: absolute temperature).

**Fig. 1:**
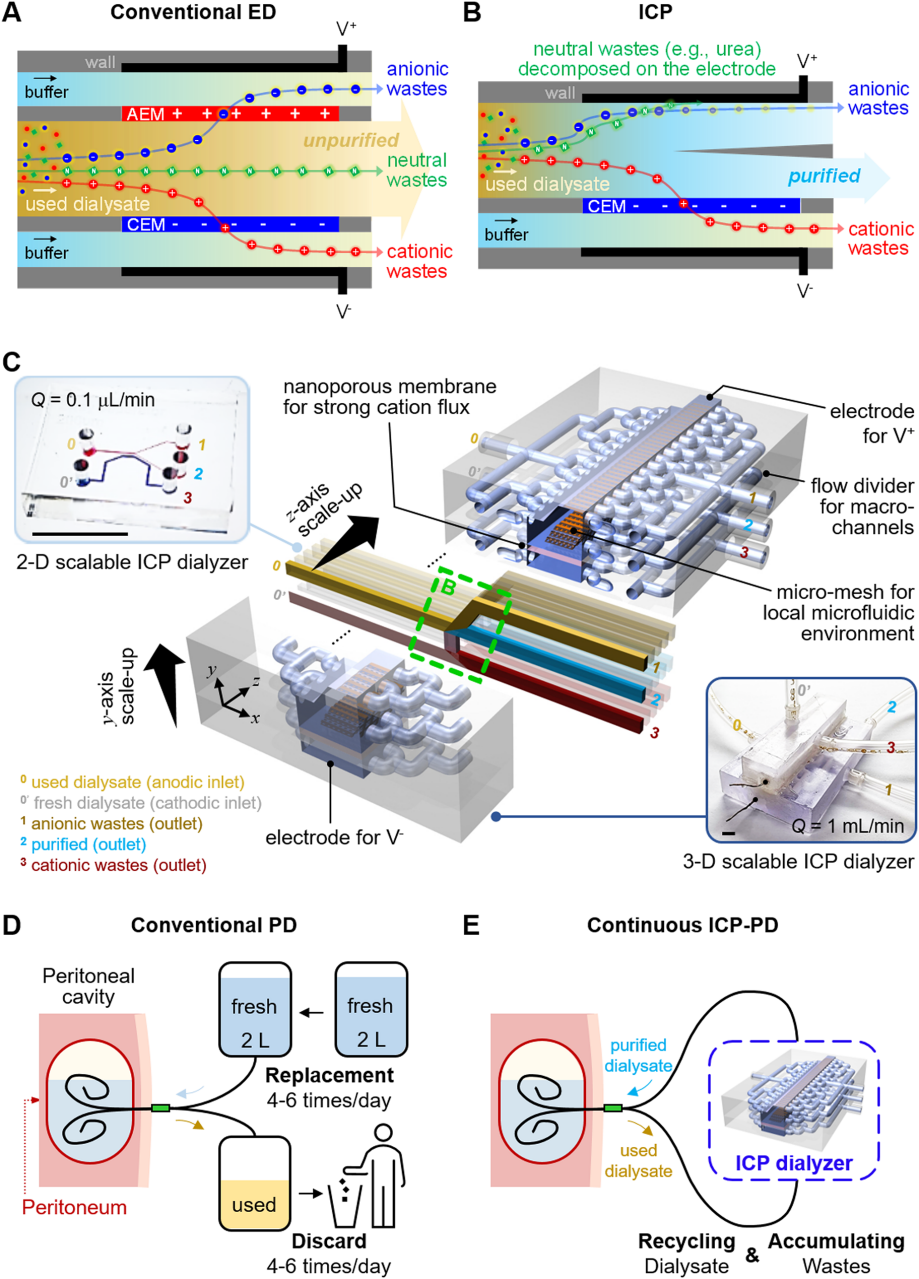
**Fig. 1. (A)** Electrodialysis (ED) is unable to remove electrically neutral compounds, as its separation mechanism relies exclusively on the use of anion exchange membrane (AEM) and cation exchange membrane (CEM), which selectively transport charged species. **(B)** Ion concentration polarization (ICP) can purify used dialysate by removing even neutral wastes. Purified dialysate would be extracted after applying a bias between the CEM, as neutrally charged urea would decompose into non-toxic gases at the anodic electrode, anionic wastes would be rerouted around the ion depletion boundary, and cationic species, including creatinine, would be removed via cationic flux through the CEM. **(C)** Schematic diagram of 3-D scalable ICP dialyzer for practical dialysate regeneration. A 2-D micro-nanofluidic hybrid dialyzer would be scaled-up to 3-D macro-fluidic dialyzer while maintaining the microfluidic environment near nanoporous membrane for practical utility. The scale bar in the inset image is 1 cm. **(D)** Schematic diagram of conventional peritoneal dialysis (PD). PD uses patient’s own peritoneum, to filter dissolved toxins in a blood with indwelled dialysate for 4 to 6 hours. **(E)** Schematic diagram of continuous PD using an ICP dialyzer capable of dialysate regeneration. The used dialysate discharged from the peritoneal cavity removed body toxins through the ICP dialyzer and was regenerated and reintroduced into the peritoneal cavity

While ED uses both cation and anion exchange membranes to remove charged components, it cannot purify neutral species because they are not affected by the electric field (Fig. 1A). Therefore, its application as a dialyzer is limited by its inability to simultaneously remove neutral urea and positively charged creatinine. Despite their merits, none of these techniques can simultaneously purify a wide size-range of target species, spanning from salt ions to biomolecular contaminants, in a single-step process. In contrast, one of the nanoelectrokinetic phenomenon, the ion concentration polarization (ICP) based purification technology [28–32], as reported recently, aligns with these criteria, owing to its distinctive electrical filtration capabilities and scalability. Briefly, the perm-selectivity of nanoporous membranes initiates an electrolyte concentration polarization on both sides of the membrane. In the case of cation-selective membranes, an ion depletion zone forms on the anodic side of the membrane [33, 34]. Charged species reroute their trajectories along the concentration gradients near this ion depletion zone, serving as a pivotal site for the purification of a broad range of contaminants.

In this study, for portable PD, we firstly proposed a scalable ICP dialysate regeneration device. ICP removes cationic components through the cation exchange membrane, anionic components by electrostatic repulsion and neutral species through an electrochemical reaction at the electrode (Fig. 1B). When urea, a neutrally charged body toxin, begins to undergo direct oxidation at the electrode inlet, the concentration of urea around the electrode decreases. The urea concentration profile exhibits a decrease closer to the electrode, and as urea diffuses towards the electrode vicinity, a chain reaction of direct oxidation occurs. As a result, a purified dialysate could be continuously obtained by extracting a stream from the ion depletion zone. Micro-nanofluidic dialysate regeneration platform was upscaled in two- and three-dimensional directions using a commercial 3-D printer as shown in Fig. 1C. The microfluidic environment within this scaled-up device was established through confined micro-geometries, preventing undesirable instabilities and enhancing the removal of cationic toxins.

Inconveniences of conventional PD (Fig. 1D) could be addressed through the introduction of a continuous ICP-PD system (Fig. 1E). This innovative system would offer efficient toxin removal, a simplified self-exchange mechanism, reduced risks of infection and metabolic complications, thus optimizing PD as an ideal renal replacement modality and capitalizing on its conceptual merits. In vitro testing utilizing used dialysate from PD patients and in vivo testing on a bilateral nephrectomy rat model were conducted to validate this innovative approach.

## Results and discussions

### 2-D scalable ion concentration polarization dialyzer

**Fig. 2:**
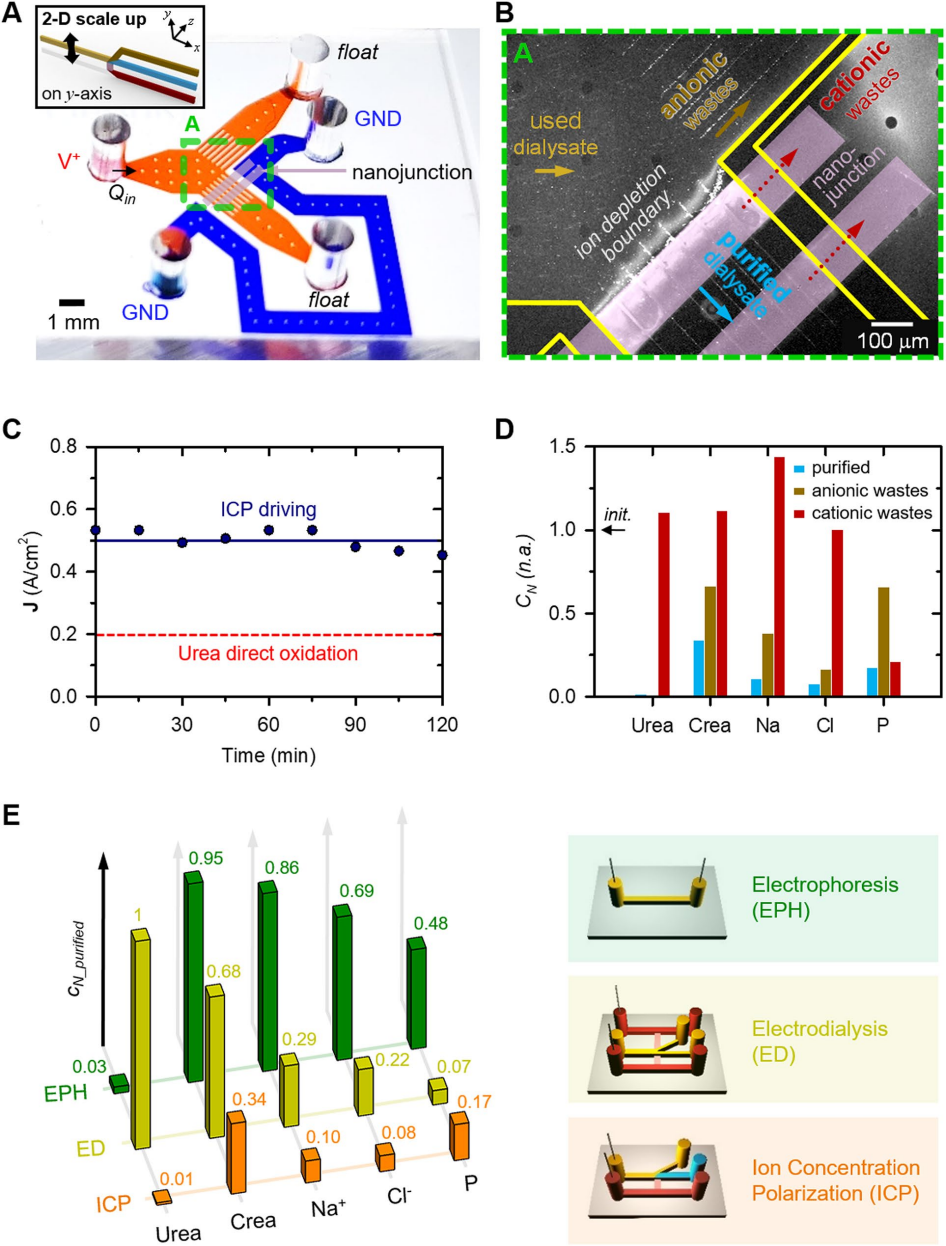
(**A**) A microscopic image of 2-D scalable ICP dialyzer. Polymeric material, PDMS was used for building microchannels and nanoporous membrane, Nafion was used for patterning nanojunctions, and the microchannel width was expanded using a micro-fin structure which suppressed an electroosmotic instability (EOI). (**B**) A microscopic image of dialysate purification due to the ICP phenomenon. Anionic wastes were rerouted outside the ion depletion boundary and cationic wastes were removed through the nanojunction by cationic flux so that purified dialysate was extracted from stream inside the ion depletion boundary. (**C**) A measurement result of current densities versus time when 70 V was applied for ICP generation. The current densities used for separation of the purified and anionic wastes streams exceeded the known current densities for urea direct oxidation [38]. (**D**) Nondimensionalized concentration profiles of major dialysis indicators inside (blue) and outside (brown) the ion depletion boundary of the anodic side streams, and the cathodic side stream (red). Concentrations of all indicators in the used dialysate decreased to below 30% in the purified channel (blue bar). (**E**) Graph showing the results of a control experiment to verify the dialysate purification mechanism. Dialysate purification was successful only for ICP condition

To demonstrate scalability of ICP dialyzer in principle, a two-dimensional (2-D) micro-nanofluidic scaling device was fabricated as shown in Fig.  2A. For details regarding its materials used, fabrication process and experimental setup, refer to the section of Experimental methods and Supplementary Information (SI) Fig.  1A. To accommodate the Renal Panel’s requirement of a minimum of 100 µL undiluted sample for measuring components in a dialysate solution, we optimized efficiency by mitigating electrokinetic instability through micro-fin structures near the bifurcation point [35] and reducing electrical resistance using double-patterned nanojunctions. Human used dialysate solution was continuously injected into both anodic and cathodic microchannels at a steady flow rate of 0.4 µL/min. Under these conditions, ICP phenomenon was generated, leading to the development of an ion depletion boundary ahead of the nanojunction. This separation mechanism utilized for wastes removal and purification, as depicted in Fig.  2B. Notably, a stable current measurement was sustained for 120 min, as demonstrated in SI Fig.  1B. Anionic fluorescent dye molecules (Alexa 488, Invitrogen, USA) and carboxylate micro-particles (1 μm diameter, Invitrogen, USA) were unable to traverse the ion depletion boundary, redirecting them outside this boundary, as indicated in SI Video 1.

In addition, Fig.  2C was incorporated to underscore that the proposed ICP dialyzer employed a current density capable of direct urea oxidation. It has been reported that, under high potentials exceeding 1.7 V versus the saturated calomel electrode (SCE), urea undergoes decomposition through a direct oxidation process at the electrode surface, leading to the formation of nitrogen and carbon dioxide gas. This phenomenon is represented by the following equations [37–39]: Eq. (1) outlines the anodic reaction, Eq. (2) delineates the cathodic reaction, and Eq. (3) represents the overall reaction.

1 $${\rm{CO}}{\left( {{\rm{N}}{{\rm{H}}_{\rm{2}}}} \right)_{\rm{2}}}{\rm{ + 6O}}{{\rm{H}}^{\rm{ - }}} \to {{\rm{N}}_{\rm{2}}}{\rm{ + 5}}{{\rm{H}}_{\rm{2}}}{\rm{O + C}}{{\rm{O}}_{\rm{2}}}{\rm{ + 6}}{{\rm{e}}^{\rm{ - }}}$$

2 $${\rm{6}}{{\rm{H}}_{\rm{2}}}{\rm{O + 6}}{{\rm{e}}^{\rm{ - }}} \to {\rm{3}}{{\rm{H}}_{\rm{2}}}{\rm{ + 6O}}{{\rm{H}}^{\rm{ - }}}$$

3 $${\rm{CO}}{\left( {{\rm{N}}{{\rm{H}}_{\rm{2}}}} \right)_{\rm{2}}}{\rm{ + }}{{\rm{H}}_{\rm{2}}}{\rm{O}} \to {{\rm{N}}_{\rm{2}}}{\rm{ + 3}}{{\rm{H}}_{\rm{2}}}{\rm{ + C}}{{\rm{O}}_{\rm{2}}}$$

Here, the electrochemical decomposition yields nitrogen and carbon dioxide gases, which are considered biologically inert with no adverse effects on human health. To provide experimental evidence supporting urea direct oxidation utilized by the ICP phenomenon, the gas-to-liquid volume ratio of N₂ and CO₂ generated from the anodic urea electrochemical decomposition reaction (1) was first calculated. When 0.29 g/mL of urea was added to fresh dialysate, the gas-to-liquid ratio was determined to be approximately 14.8–17.6%. The detailed calculation process was presented in SI Fig.  2. Subsequently, experimental validation was conducted using a device as illustrated in SI Fig.  2A. From the effluent of the anodic side channel, continuous gas production was observed as depicted in SI Fig.  2B. The gas-to-liquid volume ratio was quantified as approximately 5–8%, primarily attributed to Faradaic losses. These gas production results provided indirect evidence supporting urea direct oxidation utilized by the ICP phenomenon. Note that when urea undergoes decomposition, nitrogen oxidants are formed, which may pose a risk to human health under specific conditions: either at potentials below 1.6 V versus SCE or under natural oxidation circumstances.

Next, each stream was separately extracted, and quantifiable concentration profiles of key indicators – urea, creatinine, Na^+^, Cl^-^, and phosphorus (P) – critical for patient health assessment, were established, as shown in Fig.  2D. The concentration changes of each key indicator were nondimensionalized as follows.

$${C_N} = {\matrix{ concentration\,of\,each\,indicator \hfill \cr \,at\,the\,outlet\,({C_{indicator\_outlet}}) \hfill \cr} \over \matrix{ concentration\,of\,each\,indicator \hfill \cr \,at\,the\,inlet\,({C_{indicator\_inlet}}) \hfill \cr} }$$

First, the positively charged species (Na^+^ and creatinine) were removed from the purified stream depending on their electrophoretic mobility [36]. A substantial proportion of Na^+^ ions transited the nanojunction via cationic flux, yielding a final collection of 90% desalted stream (normalized concentration of 0.1). While creatinine (sub nanometer molecule and one of the major toxins of body wastes from a used dialysate) is neutral at pH 7.4, it acquires a positive charge under pH 7.4. Given the slightly acidic nature of the dialysate, we confirmed that creatinine follows a cationic-like transport mechanism during the ICP phenomenon development. Creatinine concentration decreased both outside (yellow channel) and inside (blue channel) the ion depletion boundary on the anodic side, but increased on the cathodic side (brown channel). Approximately 50% of creatinine crossed the nanojunction, with around 33% passing through the stream outside the ion depletion boundary. Ultimately, around 17% of creatinine remained within the ion depletion boundary, leading to a significant reduction in the purified stream concentration (normalized concentration of 0.34). Second, the negatively charged chloride ions (Cl^-^) underwent electrochemical reactions on the anodic electrode to maintain electro-neutrality due to the ICP phenomenon, causing a redistribution of concentration profiles near the nanojunction. Third, urea, an uncharged molecule and a major toxin in body wastes alongside creatinine, underwent complete elimination through electrochemical reactions in anodic side streams, including the purified stream (normalized concentrations of 0.01). Last, the weakly charged phosphorus (P) was substantially removed from both anodic and cathodic channels (normalized concentrations of 0.17). To understand the distinct removal mechanism of P compared to other components, we monitored the movement of the ion depletion boundary during a 3-hour device operation, as presented in SI Fig. 1C. The principal constituent of these precipitates was identified as phosphorus as demonstrated in SI Fig. 1D. Thus, we deduced that P did not traverse the ion depletion boundary or the nanojunction; rather, it underwent decomposition due to an electric field, subsequently being expelled through the anionic wastes channel.

As depicted in Fig.  2E, we tried to find candidates for portable peritoneal dialyzer using micro-nanofluidic devices fabricated based on three different mass transfer physics. First, we fabricated a simple microfluidic device that was governed only to electrochemical reactions and electrophoresis (referred to as “EPH” in Fig. 2E). In this setup, two electrodes were positioned within a single microchannel: one in proximity to the inlet reservoir and the other adjacent to the outlet reservoir, with no interconnecting junctions. The microchannel was loaded with used dialysate, and a combination of positive voltage and pressure was applied to the inlet reservoir. Under these conditions, complete elimination of urea was achieved, while creatinine removal reached approximately 5%, Na^+^ removal approximately 14%, Cl^-^ removal approximately 31%, and P removal approximately 52%. The decisive factor for the complete removal of urea can be attributed to an electrochemical reaction.

Second, to mimic the ED cell structure, three different microchannels and nanojunctions were constructed across them (referred to as “ED” in Fig.  2E). One microchannel was designated for anodic processes located at the upper side, another for the controlled flow of a sample along the middle path, and a third for cathodic processes situated at the lower side. Within this arrangement, it was observed that the ion depletion successfully manifested within the middle-bifurcated channel. This was due to the establishment of a robust electric field across the nanojunction between the upper anodic channel and the lower cathodic channel. Notably, urea experienced no alteration due to the absence of electrodes in the middle channel, while creatinine and Na^+^ ions were removed via electrical transport driven by the ICP phenomenon.

Finally, the conditions of ICP was employed within a micro-nanofluidic device. As mentioned earlier, the complete elimination of urea and the removal of other substances were achieved through electrical transportation utilized by the ICP phenomenon (referred to as “ICP” in Fig.  2E). The application of a lower potential hindered the removal efficiency of all components.

Based on the insights gleaned from these analyses, we concluded that the purification of dialysate can only be achieved when the ICP phenomenon was induced through the presence of an electrode within the stream of dialysate (referred to as “ICP”). We named these micro-nanofluidic devices as a 2-D scalable ICP dialyzer. In addition, by enhancing the throughput capacity from 0.1 µL/min (inset Fig. 1C) to 0.4 µL/min (Fig.  2B), we were able to conduct precise analyses by minimizing errors caused by prolonged experiments for ensuring minimum sample volume requirement of the Renal Panel measurement.

### Development of 3-D ion concentration polarization dialyzer

**Fig. 3:**
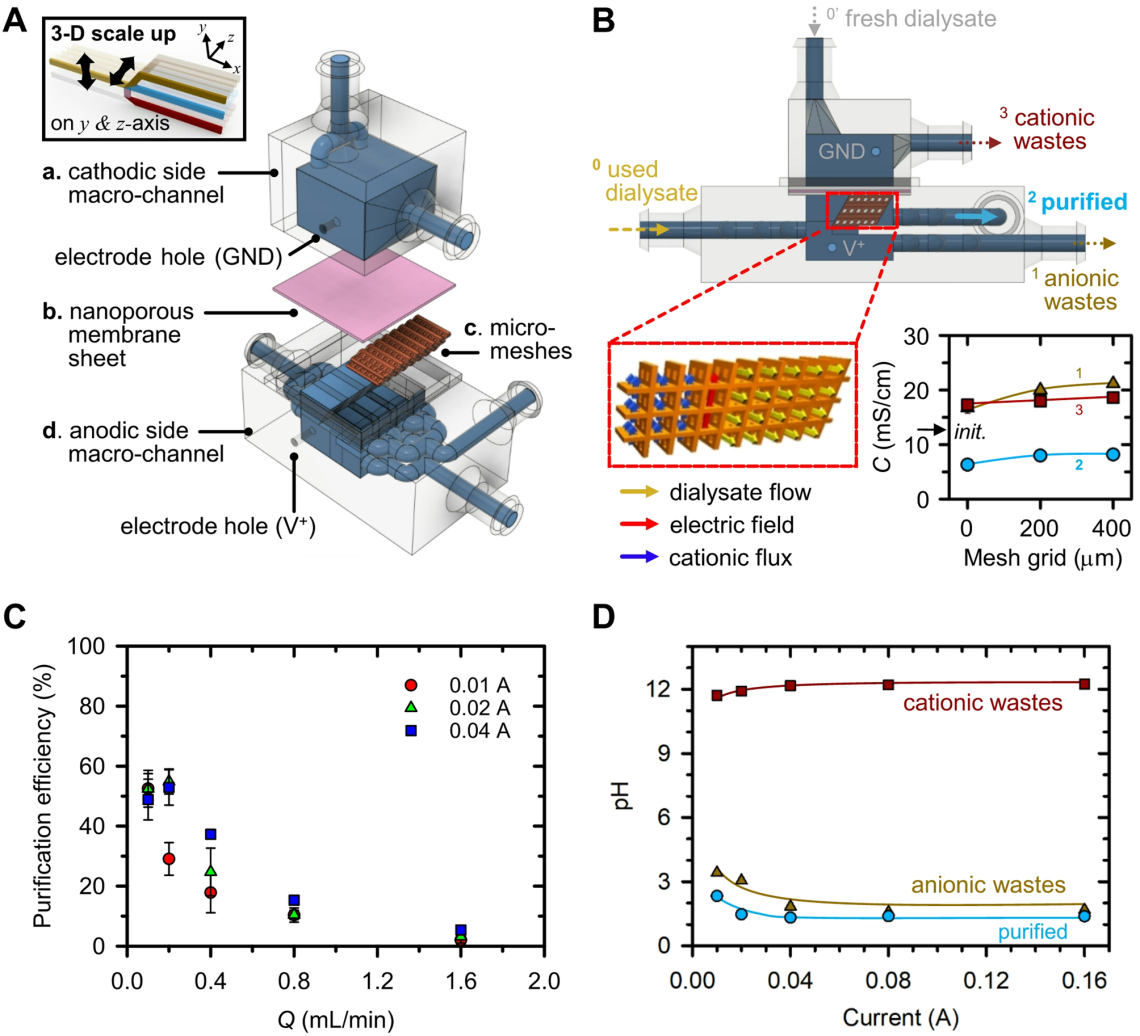
(**A**) Schematic diagram of 3-D scalable ICP dialyzer. The device consisted of a cathodic side macro-channel with inlet for fresh dialysate and outlet for cationic wastes flowing, a nanoporous membrane sheet for removing positively charged species, micro-meshes for reducing EOI and enhancing cationic flux an anodic side macro-channel with inlet for used dialysate and outlets for purified and anionic wastes flowing. (**B**) Schematic diagram and graph of micro-mesh structure design optimization. Coated nanoporous resin on the micro-meshes surface ensured that the cationic wastes were transported towards the nanoporous membrane sheet along the direction of the electric field so that purified dialysate passed through the micro-meshes perpendicular to the electric field. The micro-mesh grid size of 400 μm was chosen since the difference in conductance between purified and anionic wastes streams was the greatest, and conductance of cationic wastes was the highest. (**C)** Graph showing changes in purification efficiency according to changes in current and flow rate. Maximum purification efficiency was observed when applying *I* = 0.02 A and *Q* = 0.2 mL/min. (**D**) Graph showing changes in pH as the applied current increased under the condition of *Q* = 0.2 mL/min. As the applied current value increased, the pH of anionic wastes and purified streams decreased, and that of cationic wastes increased

While the feasibility of ICP dialyzer was demonstrated within a micro-nanofluidic hybrid configuration, the throughput of the device proved inadequate for practical dialysate recycling, either for human or animal testing purposes. In this chapter, we have engineered a macro-scale ICP dialyzer with a throughput capacity of milliliters per minute, as illustrated in Fig. 3A. This device comprises: (a) a cathodic side macro-channel, featuring an inlet for introducing fresh dialysate and an outlet for the removal of cationic wastes; (b) a nanoporous membrane sheet, serving to remove positively charged species; (c) a nanoporous resin-coated micro-mesh structure, intended to mitigate electroosmotic instability (EOI) and augment cationic flux; and (d) an anodic side macro-channel, incorporating an inlet for used dialysate and outlets for each purified and anionic wastes stream.

The fundamental concept revolved around establishing a microfluidic domain within a macro-fluidic apparatus [40]. The expansion of the ICP layer was imperative for effective dialysate filtration in this high-throughput configuration. Nonetheless, if the channel dimensions exceeded O(100) µm, analogous to other micro-nanofluidic devices, the device’s purification efficacy waned [35, 41]. ICP formation in the micro-nanofluidic regime was demarcated by three principal characteristics: (1) surface conduction (SC), (2) electroosmotic flow (EOF), and (3) electroosmotic instability (EOI), all of which exerted a pivotal influence on ion conveyance across the nanojunction [42]. Depending on the channel’s characteristic length, SC, EOF, and EOI held sway over ion transportation in very narrow channels (< 5 μm), moderately narrow channels ( < ~ O(100) µm), and wider channels ( > ~ O(100) µm), respectively. Consequently, this macroscale device was expected to exhibit EOI traits. However, it’s important to note that EOI-dominated systems inherently suffer from avoidable instability [43–45] and heightened energy consumption due to decreased overlimiting conductance [46]. To transition the system from an EOI-dominant state to EOF or SC, micro-fin structures were previously employed in a two-dimensional micro-nanofluidic setup [35]. These fins effectively curbed EOI and allowed for the integration and elevation of throughput up to levels comparable to conventional ICP devices. Nonetheless, channel expansion solely in a single plane was insufficient to achieve more than laboratory-scale throughput.

To overcome this limitation, we extended the fins in the *z*-direction (*i*.*e*., three-dimensionally), resulting in micro-meshes as depicted in Fig. 3B. This mesh was meticulously devised to generate an electric field perpendicular to the dialysate flow direction, thereby enabling cationic wastes transport along the electric field’s orientation. Additionally, an optimized micro-mesh grid size was determined through experimentation with three variants: (1) no micro-mesh, (2) 200 μm, and (3) 400 μm micro-meshes, as detailed in SI Fig. 3A. Optimal purification efficiency was realized with a micro-mesh possessing a 400 μm grid size, as this configuration exhibited the most pronounced difference in conductance between purified and anionic wastes streams. To expand the cation transport region within the macroscale device, we applied a nanoporous resin coating to the micro-mesh structure. Microscopic images of the micro-meshes before and after the nanoporous resin coating were provided in SI Fig. 3B, confirming successful coating within the open areas. The conductance results, displayed in SI Fig. 3C demonstrated the impact of the nanoporous resin coating on the micro-mesh structure. When the nanoporous resin was coated on the micro-meshes, the conductance of anionic wastes and cationic wastes streams increased, indicating enhanced cationic flux.

Subsequently, we found the optimal current and flow rate conditions that yielded the highest dialysate purification efficiency for the initial version of the 3-D scaling-up device, as depicted in Fig. 3B. The actual image of the Fig. 3B was presented in SI Fig. 3D. Due to the characteristics of the dialyzer, fluid-cell interaction occurs, making it important to use biocompatible materials [47]. Therefore, a 3-D ICP dialyzer was fabricated using biocompatible material suitable for medical applications. Materials used, fabrication and experimental details were written in the section of Experimental methods, respectively. First, as shown in SI Fig. 4A, the conductance of the purified, anionic wastes, and cationic wastes streams was measured according to changes in current and flow rate. In order to comprehensively analyze the increase in conductance due to the increase of anionic wastes in the anionic wastes stream and the decrease in conductance due to the decrease of both anionic and cationic wastes in the purified stream, purification efficiency was defined as follows and plotted in Fig. 3C.

$$\eqalign{& Purification\,efficiency \cr & = \left( {1 - {\matrix{ conductance\,of \hfill \cr purified\,stream \hfill \cr} \over \matrix{ conductance\,of \hfill \cr \,anodic\,wastes\,stream \hfill \cr} }} \right) \times 100(\% ) \cr} $$

Under the *I* = 0.01 A application, the purification efficiency decreased exponentially as the flow rate increased, and under the *I* = 0.02 A and *I* = 0.04 A application, the highest purification efficiency was obtained at *Q* = 0.2 mL/min and was relatively lower under the *I* = 0.04 A application.

Second, as shown in Fig. 3D, we confirmed the pH changes as the applied current increased under the condition of *Q* = 0.2 mL/min, which showed maximal purification efficiency. Here, we used Pt and Ag as anode and cathode electrodes material, respectively, based on the results in SI Fig. 4B. As the applied current increased, the decrease in pH of the anionic wastes and purified streams increased, and above *I* = 0.04 A, they converged and maintained a similar pH value. Meanwhile, the pH of the cationic wastes stream increased with the increase in applied current, and converged above *I* = 0.04 A. Based on the results of Fig. 3 C and D, the optimal operating conditions of the three-dimensional (3-D) ICP dialyzer were established as *I* = 0.02 A, *Q* = 0.2 mL/min, where the pH of the purified stream was relatively high and the purification efficiency was the maximum. This electrical and mechanical condition ensured a harmonious balance between high flow rate and stable purification efficiency.

### 3-D scalable ion concentration polarization dialyzer

**Fig. 4:**
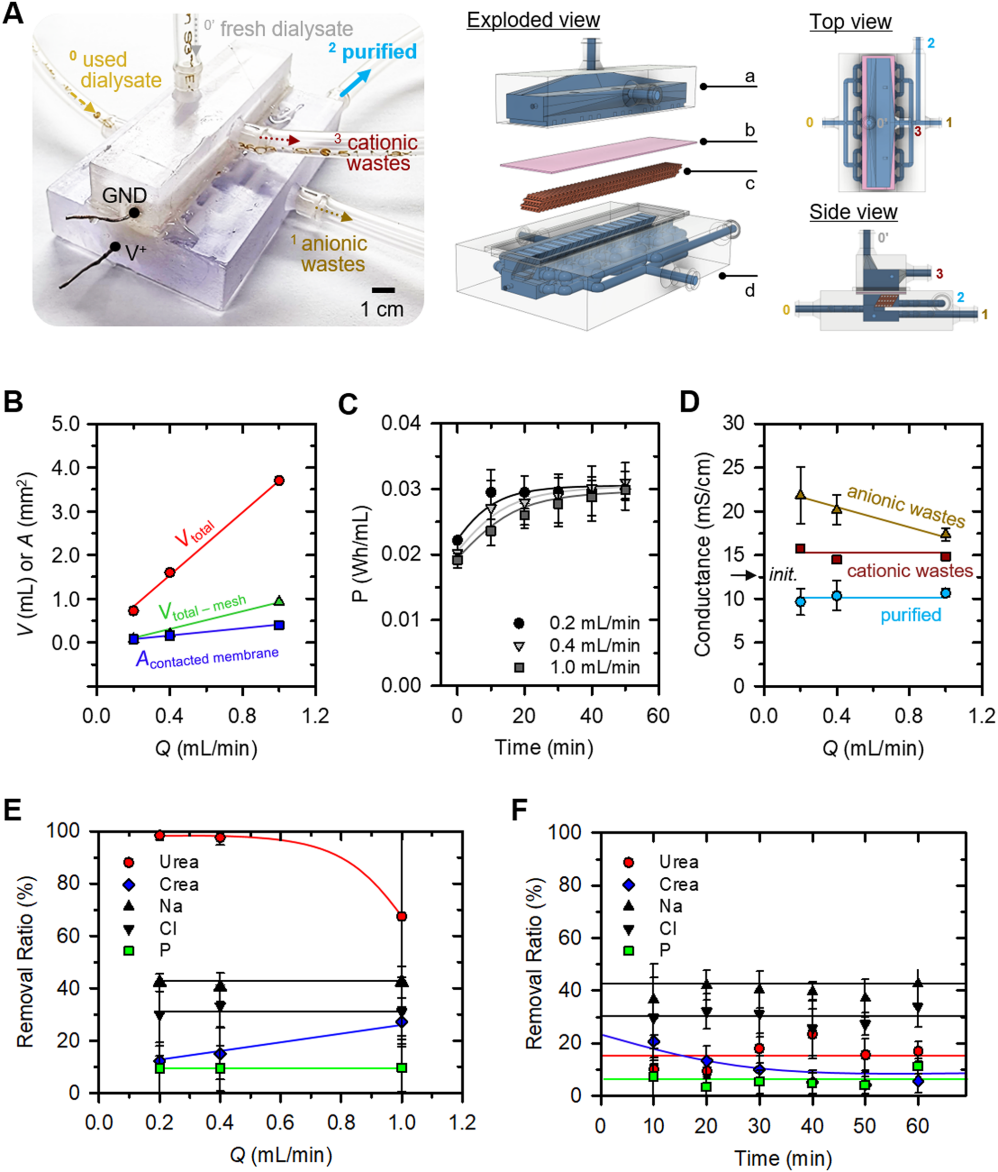
(**A**) Schematic diagram of parallelized 3-D scalable ICP dialyzer. The dialyzer was scaled up only in the y-direction from the previous design in Fig. 3(**A**) and consisted of (a) a cathodic side macro-channel, (b) a nanoporous membrane sheet, (c) a micro-mesh structure, (d) an anodic side macro-channel. (**B**) A design approach for fabricating 3-D scalable ICP dialyzer. As the throughput of the device increased, *V*_*total*_, *V*_*total-mesh*_, *A*_*contacted membrane*_ were increased proportionally. (**C**) Graph of power consumption as device throughput increases. Regardless of the throughput capacity, the power consumption increased and converged with the operation time, showing a similar tendency. (**D**) Graph of measured conductance at each channel outlets as device throughput increases. Regardless of the throughput capacity, the conductance of the purified and cationic wastes was maintained at a similar value, but the conductance of the anionic wastes channel where the electrochemical reaction occurred showed a tendency to decrease as the throughput capacity increased. (**E**) Graph of the removal ratio of the major indicators from the human used dialysate. As the throughput capacity increased, the urea removal ratio decreased, the creatinine removal ratio increased, and other ionic components showed similar values. (**F**) Graph of the removal ratio of the major indicators with respect to time using the 1 mL/min device. The removal ratio was maintained constant over time except for creatinine

The previously fabricated 3-D ICP dialyzer, which had a flow rate of *Q* = 0.2 mL/min, was further scaled up to achieve a *Q* = 1 mL/min, as shown in Fig. 4A, and an actual image of the device was shown in SI Fig. 3E. The fabrication process and assembly parts of the device were the same, only the parts size was expanded in the y-axis direction. As illustrated in Fig. 4B, the design parameters of the device were scaled proportionally in relation to the throughput increase (0.2, 0.4, and 1 mL/min), including the total volume within the device (*V*_total_), the volume excluding the mesh frame (*V*_total−mesh_), and the nanoporous membrane’s contact area (*A*_contacted membrane_) with the fluid. Based on appropriate proportional constants, a device with a maximum throughput of 1.0 mL/min was realized. Further assessment involved the power consumption of scaled devices, as shown in Fig. 4C. Although higher throughput correlated with lower power consumption, the differences were modest (within 10%), and the temporal trends remained consistent. Accumulation of gas byproducts around the anodic electrode heightened electrical resistance, with the 1 mL/min device demonstrating the lowest power consumption due to the ample outlet space for gas dispersal. Over time, the presence of trapped gases near the electrode led to increasing power consumption, eventually reaching a saturation point. Despite parallel design and manufacturing of devices, discrepancies in dialysis performance might arise.

Figure 4D showed the conductance of streams from devices with varying throughput capacities following the infusion of fresh dialysate. In the case of purified stream, the conductance exhibited a consistent decrease of around 10% compared to the introduced fresh dialysate, plateauing at approximately 10 mS/cm regardless of the flow rate (0.2, 0.4, 1 mL/min). Conversely, the conductivity of the anionic wastes stream decreased with increasing treatment capacity. This trend was attributed to reduced electrode-fluid contact area per unit capacity, resulting in decreased electrochemical reaction and wastes removal efficiency [48]. For cationic wastes stream, the conductance remained stable at approximately 15 mS/cm, irrespective of throughput capacity. This consistency was ascribed to the nanoporous membrane’s ability to maintain a uniform ion transport rate per unit area from the anodic to the cathodic channel.

Subsequent assessment involved the performance of ICP dialysis using human peritoneal dialysate, as depicted in Fig. 4E. Removal ratios for urea, creatinine, Na^+^, Cl^-^, and P were measured from purified stream. While urea’s removal ratio exhibited heightened variability with increasing throughput capacity, it maintained an average of over 50%. In contrast, the average removal ratio for anionic wastes stream was 99% (SI Fig. 5A), indicating localized decomposition of urea near the electrode. Removal ratio of Na^+^ remained consistent at around 40%, while creatinine, characterized as a weak organic cation, showed a removal ratio below 25%, which increased with higher throughput capacities. Cl^-^ exhibited average removal ratios of 20% for the 0.2 mL/min device and 25% for the 0.4 and 1 mL/min devices. P removal ratios demonstrated an increasing trend with higher throughput capacities. Concentration changes in the cationic wastes channel were presented in SI Fig. 5B, highlighting increased concentrations of Na^+^ and creatinine due to their cationic nature upon passing through the nanoporous membrane sheet.

While Fig. 4E showed the immediate measurement of collected solution upon powering on, Fig. 4F presented measurements taken from 10 to 60 min thereafter using the 3-D ICP dialyzer with *Q* = 1 mL/min. Noticeably, the removal ratio of all indicators exhibited consistent maintenance over time, barring creatinine, and urea removal ratio was dropped below 20%. In this study, we used electrode materials with the same length and surface area regardless of the device size. Urea was decomposed at the electrodes, and while bubble formation on the electrode surface was not prominent in the 0.2 mL/min device, it observably increased in the 1 mL/min device. The bubbles accumulated around the electrodes reduced the effective electrode surface area required for urea decomposition and hindered electrical paths for creatinine transportation through the nanoporous membrane. As indicated in SI Fig. 5A, the urea removal ratio remained close to 99% even in the 1 mL/min device for the anionic wastes stream, collected near the electrode, suggesting that the issue lies not with electrode performance but with device optimization. Based on these experimental evidences, we inferred that urea decomposition could be stably achieved by (1) installing a bubble removal membrane around the electrode, (2) designing the gap between the electrode and purified channel more closely, or (3) increasing the electrode surface area.

### ICP Dialyzer-assisted peritoneal Dialysis

**Fig. 5:**
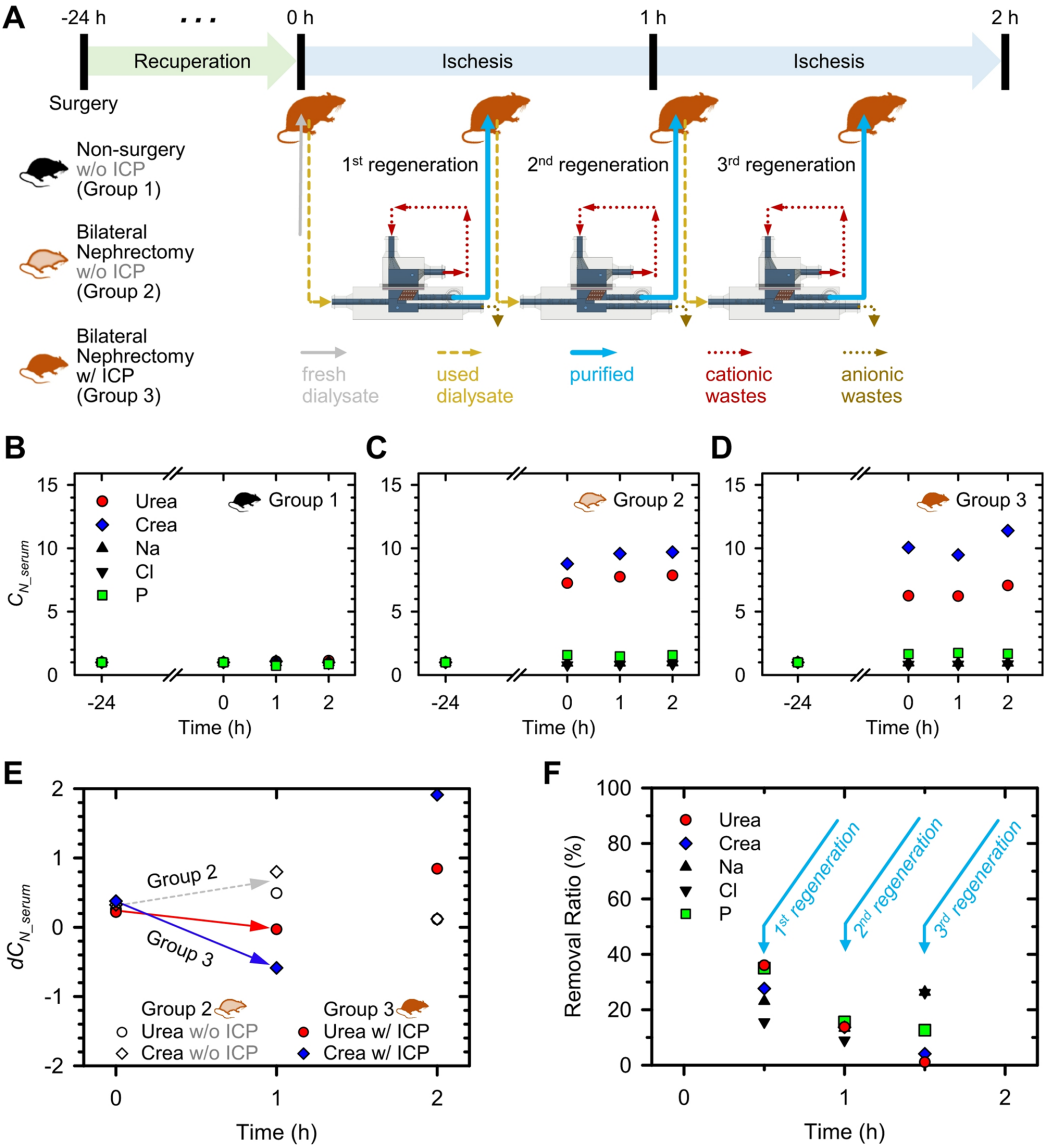
(**A**) Experimental setup for ICP dialyzer-assisted quasi-continuous PD. We aimed to investigate the in vivo toxicity changes when regenerated dialysate was reinfused at 30-minute intervals during conventional PD. (**B**) Changes in serum concentrations in group 1 (normal rats with both kidneys) when conventional PD was performed. (**C**) Changes in serum concentrations in group 2 (rat with bilateral nephrectomy) when conventional PD was performed. (**D**) Changes in serum concentrations in group 3 (rat with bilateral nephrectomy) when ICP dialyzer assisting PD was performed. (**E**) Graph showing the concentration derivatives of urea and creatinine over time for groups 2 and 3. In group 3, a noticeable reduction in in vivo toxicity was observed. (**F**) Graph showing removal ratios by ICP dialyzer over time for major indicators. A decrease in urea and creatinine clearance over time had been observed due to visible abdominal wastes

Finally, to compare the in vivo toxicity reduction performance of conventional PD and ICP dialyzer-assisted PD, we employed a bilateral nephrectomy rat model. The peritoneal cavity of the rat was too small to continuously drain the injected dialysate, so, we performed quasi-continuous PD to verify the improvement in in vivo toxicity reduction when the ICP dialyzer-assisted conventional PD, as depicted in Fig. 5A. Post-surgery, all rats were allowed a 24-hour recuperation period, followed by 2 h of respective PD sessions, during which concentrations of uremic and other pivotal components were monitored. Only for group 3 (rat with bilateral nephrectomy), after injection of fresh dialysate (0 h), 12 mL of the used dialysate was extracted and infused into the ICP dialyzer for dialysate regeneration. 4 mL of the regenerated purified dialysate was discharged and re-injected into the rat’s peritoneal cavity at 30-minute intervals.

Next, changes in serum concentrations of all major indicators were monitored and plotted, as shown in Fig. 5B for group 1, Fig. 5C for group 2, and Fig. 5D for group 3. Serum concentrations were normalized as follows.

$${C_{N_{serum}}}\left( t \right) = {{{C_{serum}}\left( t \right)} \over {{C_{serum}}\left( { - 24 h} \right)}}$$

Within the initial 24-hour period, rats with surgically removed kidneys (groups 2 and 3) exhibited a noticeable escalation in toxin concentrations, in contrast to the negligible change observed in normal rats (group 1). In groups 2 and 3, the concentrations of urea and creatinine increased sharply during the first 24 h after surgery. Following the initiation of conventional PD, the rate of increase in urea and creatinine concentrations decreased in both groups 2 and 3, while there were no significant changes in any of the indicators in group 1. To assess the rate of change in urea and creatinine concentrations in groups 2 and 3, the concentration derivatives over time were plotted in Fig. 5E. When PD was assisted by the ICP dialyzer, a distinct reduction in in vivo toxin concentrations within the first hour was observed, whereas no such reduction was achieved without the assistance. These results suggested that the ICP dialyzer could regenerate dialysate, successfully assisting PD while using a smaller dialysate volume.

However, by the second hour, an increase in in vivo toxin concentrations was observed in both groups 2 and 3. To identify the cause, we examined the removal ratios over time as shown in Fig. 5F. The results showed that the average removal ratios of urea and creatinine decreased progressively to 31.8%, 13.7%, and 2.6% for the first, second, and third infusion times, respectively. Since the used dialysate discharged from the rat’s peritoneal cavity was intermixed with various visible suspended substances, it accumulated inside the ICP dialyzer, causing non-uniformity in flow rate, electrode reactions, and electric field formation. This interaction reduced the effective surface area of the nanoporous membrane, leading to a decrease in purification efficiency over time. This effect was particularly pronounced for urea and creatinine, which are relatively larger in size compared to electrolyte ions.

## Conclusion

Currently, the sole therapeutic options for individuals with ESRD involve clinical interventions such as hemodialysis or organ transplantation. ESRD has reached a level of severity where it is even acknowledged as a malady of profound significance within the European medical context. Consequently, the advent of a portable or wearable device for PD and artificial kidney transplantation holds pivotal importance, as it has the potential to substantially ameliorate the daily existence of ESRD patients. Therefore, we anticipate that this technological innovation could serve as a beacon of respite for this cohort of patients. To pave the way for this vision, we outline a conceptual framework aimed at propelling us closer to the realization of a functional and deployable device.

A successful demonstration was conducted of 2-D and 3-D scalable ICP dialyzers designed for the peritoneal dialysate regeneration. Initially, it was confirmed that a significant reduction of toxic substances (including urea, creatinine, Na^+^, Cl^-^, and P) occurred within a micro-nanofluidic platform (see Fig.  2). Charged components such as Na^+^ and creatinine were effectively propelled through nanojunctions, governed by their electrophoretic mobility, while electrically neutral substances like urea underwent electrochemical decomposition when exposed to potentials above certain thresholds. To extend these effective elimination mechanisms into a larger-scale fluidic environment, a meticulous design approach was employed to create upscaled devices using 3-D printing techniques, incorporating a fine micro-mesh structure and a nanoporous membrane sheet (see Fig. 3). Finally, we achieved the capability to regenerate peritoneal used dialysate at a flow rate of 1 mL/min (10,000-fold enhanced from micro-nanofluidic hybrid dialyzer), resulting in a removal ratio around 30% (see Fig. 4). Additionally, through in vivo experiments with a bilateral nephrectomy rat model, we demonstrated that regenerated dialysate from the ICP dialyzer successfully assisted PD by reducing in vivo toxicity and saving dialysate volume (see Fig. 5).

In summation, the synthesis of the diverse scientific evidence we have put forth substantiates the envisaged viability of harnessing a portable peritoneal dialysis apparatus. Through the integration of proactive filtration mechanisms, real-time monitoring sensors, power provisioning units, and nutritional delivery systems, a paradigm-shifting device with the potential to effectively reinstate the quotidian functioning of individuals afflicted by ESRD is anticipated to be realized.

## Experimental methods

### Fabrication of micro-nanofluidic devices

A micro-nanofluidic device was constructed, comprising a bifurcated microchannel on the anodic side, a nanojunction, and a singular microchannel on the cathodic side. The fabrication involved employing a Polydimethyl siloxane (PDMS, Sylgard 184 Silicone elastomer kit, Dow Corning) substrate and a Nafion polymeric solution (20 w.t.% resin, Sigma Aldrich). Initially, both the anodic and cathodic microchannels were designed to possess dimensions of 1 mm in width and 15 μm in depth, to establish fluidic pathways. A mixture of PDMS pre-polymer and curing agent, in a ratio of 10:1, was de-aerated in a vacuum chamber for approximately an hour. Subsequently, the polymer mixture was poured onto a master containing microchannel patterns and cured in an oven for approximately 4 h. For the creation of the nanojunction, a Nafion solution was employed as a cationic perm-selective nanoporous substance. This solution was patterned between the bifurcation point of the anodic microchannel and the cathodic microchannel using a surface patterning technique [46, 49, 50]. Finally, precise alignment and chemical bonding of the PDMS block, housing the fluidic channels, and the glass slide containing the solid Nafion nanojunction were achieved using a plasma bonder (Cute-MP, Femto Science, Korea). The resulting assembled device was depicted in Fig.  2A.

### Apparatus for micro-nanofluidic experiment

The induction of asymmetric ion depletion boundaries necessitated the involvement of both external voltage and pressure sources. An external voltage was implemented across the nanojunction with the utilization of a source measurement unit (SMU 238, Keithley, USA). The introduction of analyte and buffer solutions into the microchannel was achieved through the utilization of a syringe pump (PHD 2000, Harvard Apparatus, USA). The detection and visualization of the electrokinetic flow within the microchannel were executed using an inverted fluorescence microscope (IX-51, Olympus, Japan) coupled with a CCD camera (DP73, Olympus, Japan). The coordination of the CCD camera with the microscope and subsequent image analysis were carried out using commercial software (CellSense, Olympus). The constituents within a dialysate were assessed utilizing a Renal Panel (7180 Hitachi Automatic Analyzer, Japan). For the experiment in Fig.  2, a flow rate of 0.4 µL/min was applied to each of the single inlets on the anodic and cathodic sides using a syringe pump. It was confirmed that similar flow rates were discharged from the two outlets on the anodic side, which were designed with identical fluidic resistance. Although the two outlet branch channels were manufactured with the same dimensions to ensure equal fluidic resistance, there might be slight discrepancies due to manually punching the outlet reservoirs with a biopsy punch. Therefore, the flow rates for the inlet, anionic wastes outlet, and purified outlet were 0.4 µL/min, 0.2 µL/min, and 0.2 µL/min, respectively.

### Building a 3-D scalable ICP dialyzer

The design of the 3-D scalable ICP dialyzers was created using a commercial 3-D drawing software (RhinoCeros 5.0). These devices consisted of distinct components including anodic compartments, a micro-mesh structure, a nanoporous membrane, and cathodic compartments, all illustrated in Fig. 3A. Here Nafion nanoporous membrane sheet (with a thickness of 0.002 inches, sourced from Sigma Aldrich) and a Nafion polymeric solution (20 w.t.% resin, Sigma Aldrich) were used in this work. The channel frames were built using a Projet 2500 + 3-D printer from 3Dsystems, USA, utilizing M2R-CL resin. M2R-CL resin is an ISO 10993 biocompatibility certified material with a dielectric constant of 3.15 at 1 MHz.

### Apparatus for macro-fluidic experiment

The process of device assembly involved the use of non-toxic silicon adhesive as a bonding agent. The interconnections between the device and the in vitro circuit were established through the utilization of Tigon tubes with an inner diameter of 3.2 millimeters, specifically sourced from ISMATEC. To use the necessary electrochemical processes, an external voltage was meticulously applied, a task accomplished through employment of a source measurement unit, namely the Lambda zup 36 − 6 from TDK. The controlled injection and suction of the analyte (human used dialysate from Seoul National University Hospital) and buffer solutions (fresh dialysate from BAXTER, USA) was achieved using a syringe pump (PHD 2000, Harvard Apparatus, USA). Subsequently, distinct streams of the injected solutions were extracted and isolated for analysis. Concentration profiles of specific ions including Na^+^ and Cl^−^, along with analytes like creatinine and urea, were quantitatively assessed using the Renal Panel method by the HITACHI 7180 instrument.

## Electronic supplementary material

Below is the link to the electronic supplementary material.

Supplementary Material 1

Supplementary Material 2

## Data Availability

No datasets were generated or analysed during the current study.
