# Supplementary material for: Scalable ion concentration polarization dialyzer for peritoneal dialysate regeneration
Source: J Nanobiotechnology. 2025 Mar 29;23:255. doi: 10.1186/s12951-025-03294-1 (PMC11954356; doi:10.1186/s12951-025-03294-1)
Supplement: Supplementary file 2 — Supplementary Material 2 [file 12951_2025_3294_MOESM2_ESM.docx]

Supporting information for

Scalable Ion Concentration Polarization Dialyzer

for Peritoneal Dialysate Regeneration

1. Micro-nanofluidic Experiment Details

SI Figure 1. A. 2-D micro-nanofluidic device and experimental set-up on the microscope. B. Current measurement for 120 minutes under ICP operation. C. Long-term operating images of ICP phenomenon. Precipitates were formed near the ion depletion. D. A measurement result of the precipitates. The main component of the precipitates was phosphorus.

An experimental setup of two-dimensional (2-D) micro-nanofluidic scaling device on the microscope was shown in SI Figure 1A. Particle behavior in the device was imaged using a fluorescence inverted microscope, and samples were continuously collected by connecting a tube to the anodic wastes and purified channel outlets. During the constant voltage application (70 V), stably maintained current was measured for 120 minutes, as demonstrated in SI Figure 1B. To understand the distinct removal mechanism of P compared to other components, we monitored the movement of the ion depletion boundary during a 3-hour device operation, as presented in SI Figure 1C. This analysis confirmed the formation of precipitates near the ion depletion boundary and in the outer (anodic wastes) channel after approximately 2 hours of operation. As demonstrated in SI Figure 1D, the principal constituent of these precipitates was identified as phosphorus. Thus, we deduced that phosphorus did not traverse the ion depletion boundary or the nanojunction; rather, it underwent decomposition due to an electric field, subsequently being expelled through the anodic wastes channel.

**2. Calculation and experimental evidence for electrochemical decomposition of urea**

SI Figure 2. A. A macro-scale ICP operating device (*Q* = 1 mL/min) developed to quantify the gas-to-liquid volume ratio generated by the electrochemical decomposition of urea at the anode. B. The gas volume observable in the effluent line of the anodic side channel.

The urea decomposition products generated through electrochemical reactions at the anode, as reported in the literature [*Electrochimica Acta* 2007, **52**, 5696], include CO₂, N₂, H₂, CNO⁻, NO₃⁻, NO₂⁻, [N₂O₂]⁻, N₂O, NO₂, and NH₂CONH₂•⁺. The specific decomposition products are influenced by the applied potential. Under the current density conditions employed in this study (refer to Figure 2C), it has been established that the direct oxidation of urea (CO(NH₂)₂) occurs, resulting in its decomposition into carbon dioxide (CO₂) and nitrogen (N₂).

The electrochemical reaction equation at the anode: CO(NH₂)₂ + 6OH⁻ → N₂ + 5H₂O + CO₂ + 6e⁻ was utilized to calculate the carbon dioxide and nitrogen gas generation ratio by urea decomposition. In this electrode reaction of urea, one mole of nitrogen and one mole of carbon dioxide will be produced from one mole of urea. Carbon dioxide generated during the process dissolves in water at a rate of 50-70 %, while 30-50 % remains in the gaseous state. Additionally, it undergoes partial ionization to form hydrogen ions (H⁺) and bicarbonate ions (HCO₃⁻), though the extent of ionization is relatively low. In contrast, nitrogen is only 1-5 % dissolved in water, with 95-99 % remaining in a gaseous state. At standard temperature and pressure (STP; 0°C, 1 atm), the molar volume of gas is 22.4 L/mol, while under the experimental conditions of atmospheric pressure and room temperature (25 °C), the molar volume is 24.45 L/mol. Upon the electrolysis of 1 mole of urea, the gas production from carbon dioxide is calculated to range between 7.335 L (24.45 × 0.3) and 12.225 L (24.45 × 0.5). Similarly, the gas generation from 1 mole of nitrogen is estimated to range between 23.228 L (24.45 × 0.95) and 24.206 L (24.45 × 0.99). Therefore, the total volume of gas produced is determined to be within the range of 30.563 to 36.431 L.

The urea concentration in the peritoneal dialysis solution used in this study was 0.29 g/L, corresponding to 4.83 × 10⁻³ M. Given that 1 mole of urea produces a gas volume ranging from 30.563 to 36.431 L, the urea concentration of 4.83 × 10⁻³ M is expected to generate between 0.148 L (30.563 × 4.83 × 10⁻³) and 0.176 L (36.431 × 4.83 × 10⁻³) of gas. Thus, for 1 L of peritoneal dialysis solution, 0.148–0.176 L of gas is generated. This corresponds to approximately 14.8–17.6 % of the total liquid volume being converted into gas at the outlet. Subsequently, to experimentally validate the gas-to-liquid volume ratio resulting from urea electrochemical decomposition at the anode, a device with a throughput capacity of ***Q* = 1 mL/min** was designed, as illustrated in **SI Figure 2A.** This device featured independent anodic and cathodic side channels separated by a Nafion nanoporous membrane. Upon applying voltage (20 V) and flow rate (1 mL/min), the experimentally determined gas-to-liquid volume ratio was approximately 5-8 %, as shown in **SI Figure 2B.**

The Faradaic efficiency ($\eta_{F}=\frac{Observed amount of product (L)}{Theoretical amount of product (L)}\times100 (\%)$) can be calculated to evaluate the correlation between the volume of gas produced and the actual charge consumed. Based on a comparison between the theoretically predicted values and the experimentally obtained results, the Faradaic efficiency was estimated to range from 28.4 to 54.1 %. The lowered Faradaic efficiency suggested that the device's channel and electrode configurations had not been fully optimized. This result indicated the occurrence of Faradaic losses due to thermodynamic irreversibility. Furthermore, during the experiment, a portion of the gas generated at the electrode failed to exit through the outlet tube and instead accumulated within the device. This limitation appears to be associated with a design that predominantly considers inertial forces. Since the gas produced during urea decomposition had a lower density compared to the dialysis solution, it was subject to both inertial and gravitational forces. Consequently, the reduction of the dialysis solution-electrode interface minimized the active reaction sites for electrolysis, potentially leading to the occurrence of side reactions rather than efficient gas generation.

**3. Fabrication of 3-D Scalable Ion Concentration Polarization Dialyzer**

SI Figure 3. A. 3-D printed micro-meshes with different grid size of 200 μm and 400 μm. B. Microscopic images of the micro-meshes with and without Nafion 20 w.t.% coating. C. Conductance measurement results of purified, anodic and cathodic wastes streams depending on the presence or absence of Nafion coating on the mesh. D. An image of fabricated 3-D scalable ICP dialyzer with 0.2 mL/min, and E. 1.0 mL/min throughput capacity.

SI Figure 3A showed 3-D printed mesh structures with grid size of 200 μm and 400 μm, respectively. The grid size was adjustable and 400 μm was chosen in this work based on control experiment. We performed a Nafion coating on the mesh in order to enhance the mobility of cationic ions in the 3-D scalable ICP dialyzer. SI Figure 3B showed a microscopic image of the mesh before and after the Nafion coating, respectively. These results confirmed the presence of coagulated Nafion on the open channels. Coated Nafion on the mesh surface ensured that the positively charged species were transported towards the Nafion sheet along the direction of the electric field. Therefore, cationic wastes in a used dialysate were removed through Nafion-coated mesh and Nafion sheet along with applied electric field direction, and purified dialysate passed through the mesh perpendicular to the electric field. SI Figure 3C showed the conductance measurement results of purified, anodic wastes, and cathodic wastes streams depending on the presence or absence of Nafion coating on the mesh. The conductance difference according to the presence or absence of the mesh coating was difficult to confirm significantly in the purified stream, but it was confirmed that it was improved by about 10 % in the anodic and cathodic wastes streams. SI Figure 3D and 3E present actual photographs of the fabricated 3-D scalable ICP dialyzer with throughput capacity of 0.2 mL/min and 1.0 mL/min, respectively. The 1.0 mL/min ICP dialyzer was fabricated based on the 0.2 mL/min device structure, and parallel scaling-up was performed especially considering *V*_total_, *V*_total-mesh_, and *A*_contacted membrane_ (as explained in Figure 4B).

4. Optimization of 3-D Scalable Ion Concentration Polarization Dialyzer

SI Figure 4. A. Conductance measurement results of purified, anodic and cathodic wastes streams to optimize flow rate and current source application condition for the 3-D scalable ICP dialyzer. B. The pH measurement results of purified, anodic and cathodic wastes streams with different electrodes materials.

To optimize flow rate and current source application condition for the 3-D scalable ICP dialyzer depicted in Figure 3A and SI Figure 3D, conductance of purified, anodic wastes, and cathodic wastes streams was measured as shown in SI Figure 4A. Currents of 0.01, 0.02, and 0.04 A were applied to the 3-D ICP dialyzer, while fresh dialysate was injected into the anodic and cathodic side channels at flow rates of 0.1, 0.2, 0.4, 0.8, and 1.6 mL/min for each case. At the slowest flow rate of 0.2 mL/min, there was a large difference in conductance between anodic wastes and purified streams, and there was almost no difference at flow rates of 0.4 mL/min or higher. As shown in SI Figure 4B, we measured the pH changes as the applied current increased under the condition of *Q* = 0.2 mL/min with different electrodes materials. When Ag was used as the anode, the amount of bubbles generated by the electrode reaction was quite large, and above 0.02A, the channel was blocked and no discharged streams could be obtained. The pH value of the streams was the same when both the anode and cathode were used as Pt and when the anode was used as Pt and the cathode as Ag. Therefore, the latter was chosen as the electrode materials because it has the advantage of cost reduction.

5. Toxin and Electrolyte Ions Removal in the Anodic and Cathodic Wastes Channels

SI Figure 5. Toxin and electrolyte ions removal ratios of 3-D ICP dialyzer in the A. anodic and B. cathodic wastes channels

For the 3-D ICP dialyzer depicted in Figure 4A and SI Figure 3E, toxin and electrolyte removal ratios in the anodic and cathodic wastes channels were presented in SI Figure 5A and 5B, respectively. In the anodic wastes channel, the removal ratio of urea remained the same for 60 minutes at a level of 99 %, while the removal ratio of other electrolyte components was less than 25 %. Since the anodic wastes channel was adjacent to the electrode, the removal of urea eliminated by the electrochemical reaction was the largest. On the other hand, the removal ratio of ions was measured lower than that of the purified channel (result in Figure 4F) because the anodic wastes channel was far from the Nafion sheet where cation transport occurs. In SI Figure 5B, a negative removal ratio means an increase in concentration. Concentrations of positively charged Na^+^ and creatinine were measured at levels above 25 %. Since the creatinine concentration initially injected into the cathodic channel was close to zero, the increase ratio was highly measured.
